# Picophytoplankton in the West Pacific Ocean: A Snapshot

**DOI:** 10.3389/fmicb.2022.811227

**Published:** 2022-03-22

**Authors:** Feng Wang, Yuqiu Wei, Guicheng Zhang, Linlin Zhang, Jun Sun

**Affiliations:** ^1^State Key Laboratory of Biogeology and Environmental Geology, China University of Geosciences (Wuhan), Wuhan, China; ^2^College of Marine Science and Technology, China University of Geosciences (Wuhan), Wuhan, China; ^3^Research Centre for Indian Ocean Ecosystem, Tianjin University of Science and Technology, Tianjin, China; ^4^Key Laboratory of Ocean Circulation and Waves, Institute of Oceanology, Chinese Academy of Sciences, Qingdao, China; ^5^Yellow Sea Fisheries Research Institute, Chinese Academy of Fishery Sciences, Qingdao, China; ^6^Pilot National Laboratory for Marine Science and Technology (Qingdao), Qingdao, China

**Keywords:** picophytoplankton, carbon-to-Chlorophyll *a*, environmental factors, Western Pacific Ocean, abundance

## Abstract

Marine picophytoplankton have crucial ecological value and make an important contribution to marine primary productivity. While biomass of phytoplankton in general is projected to decline as a result of global warming, picophytoplankton will likely dominate in the future oceans due to their growth advantages in an oligotrophic environment. To better understand the biography of picophytoplankton, we undertook a comprehensive study of the distribution patterns of picophytoplankton, carbon biomass, and Chl *a* concentrations, etc. based on large-scale sampling in the tropical Western Pacific Ocean. In terms of cellular abundance, *Prochlorococcus* was the most abundant group (averaging [1.03 ± 0.40] × 10^4^ cells/mL), followed by *Synechococcus* (averaging [1.31 ± 1.22] × 10^3^ cells/mL) and then picoeucaryote (averaging [4.83 ± 2.84] × 10^2^ cells/mL). The picophytoplankton size-fractionated chlorophyll *a* (Pico-Chl *a*) accounted for about 30% of the total Chl *a*, with *Prochlorococcus* and picoeukaryotes contributing 41 and 35%, respectively, of the Pico-Chl *a*-normalized carbon biomass, indicating the ecological importance of picophytoplankton as the primary producers. In terms of biogeographic distribution, the picophytoplankton communities exhibited contrasting patterns. The surface distribution of *Prochlorococcus* and *Synechococcus* was concentrated in the low latitude of the 142°E section, while picoeucaryote was more abundant near the 130°E and equator sections. *Synechococcus* was higher in the shallow layer at 25 m, and it was extremely tolerant of high-light irradiation, while *Prochlorococcus* and picoeucaryote were distributed in the deep Chlorophyll maximum layer (DCM) (about 100 m). From the carbon-to-Chlorophyll *a* ratios, which was derived from *Prochlorococcus* and picoeucaryote population groups, we found that the ratio varied widely, from 0.19 to 75.56, and was highest at the depth of 200 m. Of these, *Prochlorococcus* had an important contribution. The correlation analysis of environmental factors showed that *Prochlorococcus*, *Synechococcus*, and picoeucaryote were negatively correlated with nutrient concentration. We concluded that *Prochlorococcus* group was dominant in the WPO, both in abundance and biomass, and the various abiotic factors such as temperature, salinity, and nutrient concentrations were closely correlated with the spatial variation in the picophytoplankton community. These findings aid our understanding of how contrasting environmental conditions influence picophytoplankton community and the importance of picophytoplankton in contributing the carbon pool in the oligotrophic ocean.

## Introduction

Picophytoplankton (<2μm), including *Synechococcus*, *Prochlorococcus*, and the picoeucaryotes, are of great significance to the marine ecosystem. The first two of these taxa are cyanobacteria and belong to autotrophic prokaryotes. Picophytoplankton are ubiquitous in freshwater and aquatic environments, and are even found in the polar regions (Wright et al., 2009). Compared with micro- or nano-phytoplankton, picophytoplankton are more efficient at obtaining and utilizing nutrients to promote growth and reproduction. Under future, warmer ocean conditions, where warming-induced stratification blocks nutrient supply, picophytoplankton biomass will increase along a temperature gradient in low-latitude regions (Taniguchi et al., 2014; Flombaum et al., 2020). Moreover, the growth rate of picophytoplankton is fast, which is adaptive to the high mortality rates caused by microzooplankton grazing. Through photosynthesis by the picophytoplankton community, CO_2_ in the atmosphere is transformed into dissolved organic carbon (DOC) or particulate organic carbon (POC), which is then transported to the deep sea through a series of processes and finally buried in seabed sediments, resulting in a reduction in the concentration of CO_2_ in the atmosphere (Longhurst and Harrison, 1989). It is reported that picophytoplankton contribute up to 80% of the fixed carbon in some oligotrophic regions (Liang et al., 2017). Ning et al. (1996) showed that Chl *a* specific productivity of picophytoplankton contribute the most to the total productivity of high-latitude regions, and its high photosynthesis assimilation demonstrates its importance in the marine ecosystems. Furthermore, picophytoplankton contribute more to biomass and primary productivity in oligotrophic conditions or low-light environments, accounting for at least 10% of the net primary productivity of the global ocean (Raven, 1998).

The Pacific Ocean is the largest and deepest ocean in the world, with many marginal seas and islands, and its geography is complex (Yan et al., 1992). Under the movement of the current from east to west, warm seawater is brought to the Western Pacific Ocean (WPO) (Zhang et al., 2012). In addition, the equatorial region has strong light radiation all year round, which can obviously heat seawater. Therefore, the surface water is higher than 28°C (Hu, 2016). Not only perennial tropical storms and typhoons have brought a lot of rainfall to this region, but also the permanent thermocline prevents the bottom nutrient-rich water from upwelling to the surface layer, reducing the surface nutrient concentration (Kawahata et al., 2002). These environmental factors have caused the WPO to become a typical oligotrophic area. WPO is the region with the strongest sea-atmosphere interaction in the world, and it is also a source of continuous release of latent heat into the atmosphere (Hu et al., 2020). Heat transferring between the sea and the air will cause global climate variation. Complexity of the tropical WPO results from its three-dimensional circulation system and strong multi-scale variability, making it a vital component of the hydrologic circulation and energy cycle. The complex ocean circulation system and dense multi-scale air-sea interaction in the tropical WPO make it unique and important in the multi-scale changes of the marine environment, water mass exchange, nutrient transport, and climate events. High temperature, low salinity, and oligotrophic and variable circulation systems make the Western Pacific a hot spot for studying various ecological and environmental issues.

A great deal of information has demonstrated that picophytoplankton abundance is high in the WPO, despite very low nutrient concentrations (Fuminori et al., 2014; Agusti et al., 2019). Picophytoplankton represent on average 47% of total carbon biomass compared to diatoms (∼15%) and is approximately 4∼5 orders of magnitude higher in abundance than are diatoms (Wei et al., 2021). Furthermore, analyses of satellite-derived phytoplankton concentrations have suggested that global phytoplankton concentration has declined over the past century (Boyce et al., 2011). Earth system models predicate that ocean phytoplankton biomass has decreased, but picophytoplankton biomass would increase in low-latitude regions (Flombaum et al., 2020). Fully aware of the importance of picophytoplankton, we set up our research objectives focusing on the biology of the picophytoplankton community, including cell abundance, carbon biomass, size-fractionated chlorophyll *a* (> 20 μm, 2∼20 μm, and < 2 μm) and carbon-to-Chlorophyll *a* ratios of the picophytoplankton community in the WPO. Here, we roughly calculate the carbon biomass of the picophytoplankton community by conversion from cell abundances (Campbell et al., 1997), in order to provide references for the future study of the status of the picophytoplankton community within the marine carbon cycle. Understanding the carbon biomass of picophytoplankton is important for assessing their relative contribution to the oceanic biomass. At present, the remote determination of phytoplankton carbon biomass has proven elusive (Behrenfeld et al., 2005). A commonly used method for estimating phytoplankton biomass is to assume a given cell size and rely on some universal carbon-to-volume ratios derived from the literature to estimate the picophytoplankton carbon biomass. But for different sea areas, the carbon conversion coefficients of different picophytoplankton groups vary. On the other hand, we have the consensus that Chlorophyll *a* (Chl *a*) is the most common method for characterizing the abundance of phytoplankton (Taylor et al., 1997). Much research has been done to assess biomass and primary productivity using remotely sensed Chl *a* concentrations (Moore and Abbott, 2000). More importantly, there have been a number of attempts to calculate the relationship between phytoplankton carbon biomass and chlorophyll *a* (Sathyendranath et al., 2009). In the present study, we undertake a preliminary investigation into the ratio of carbon to chlorophyll *a*, in order to understand the picophytoplankton community from multiple perspectives. The goal of our study is to explore the spatial variation of the picophytoplankton community in the oligotrophic ecosystem, as well as estimate the contribution of picophytoplankton to the carbon biomass and primary productivity within the WPO. In addition, we have studied the relationship between picophytoplankton and various hydrological (temperature, salinity) and chemical (nutrient) conditions.

## Materials and Methods

### Study Area and Sample Collection

**FIGURE 1:**
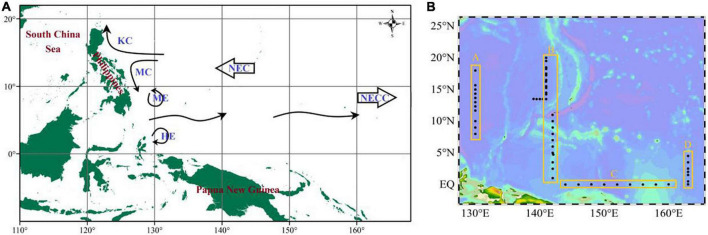
Schematic showing the circulations **(A)** and sampling stations **(B)** for the cruise WPO. KC, Kuroshio current; MC, Mindanao current; ME, Mindanao Eddy; HE, Halmahera Eddy; NEC, North Equational current; NECC, North Equational counter current.

This survey was undertaken in the Philippines to the west and in Papua New Guinea to the south. Circulations had perennial impacts on the marine ecological environment in the region (Figure 1A). Our data were obtained on the R/V Kexue cruise from October 2019 to January 2020 in the Western Pacific Ocean (0°N∼20°N, 130°E∼165°E). About sixty stations were investigated and were assigned to transect A (130°E), B (142°E), C (EQ), and D (165°E) (Figure 1B).

At each station, seawater samples were taken at up to seven depths within the upper 200 m of the water column using 12-L Niskin bottles equipped with a SeaBird CTD (SBE 19 Plus) rosette sampler. Physiochemical parameters such as salinity, temperature, and depth were also recorded *in situ* at the same time using the CTD sensors. For nutrients analysis, 100 mL of seawater was collected and because there were fewer particles matters in the open ocean, unfiltered *in situ* seawater was directly collected. This was immediately refrigerated at −20°C for further analysis. Samples for flow cytometry (FCM) analysis were directly pipetted from *in situ* seawater and fixed with paraformaldehyde (final concentration: 1%). The samples were then left to stand for 10∼15 min in the dark at room temperature, so as not to lose resolution or affect cell counting due to fixation or solidification. They were then immediately frozen in liquid nitrogen until delivery to the laboratory (Marie et al., 1997). To obtain a sample of the size-fractionated Chl *a*, 1L of seawater was filtered through three successive in-line filter holders (20 mm diameter) with a 20 μm pore size silk net followed by a 2 μm pore size nylon membrane and then 0.2 μm pore size polycarbonate filters under a filtration vacuum of less than 100 mm Hg. The particle sizes > 20, 2∼20, and 0.7∼2 μm, respectively, represent micro-, nano- and pico-Chl *a*. The filter membranes were collected and then refrigerated at −20°C for further analysis.

### Sample Analysis

#### Determination of Dissolved Nutrients

Samples of nutrients were thawed at room temperature, then were used to determine the concentration. Analyses of the nutrient concentrations including DIN (Dissolved Inorganic Nitrogen, DIN:NO_2_^–^ + NO_3_^–^ + NH_4_^+^), phosphate and silicate were performed using a Technicon AA3 Auto-Analyzer (Bran + Luebbe), following classical colorimetric methods. For determining phosphorus (PO_4_-P) with phosphor molybdenum blue spectrophotometry, the detection limit was set at 0.02 μmol/L; Determination of dissolved silicate (SiO_3_-Si) using the silicon molybdenum blue spectrophotometry involved a detection limit was 0.02 μmol/L. Determination of nitrate (NO_3_-N) using cadmium-copper column reduction involved a detection limit of 0.01 μmol/L. Determination of nitrite (NO_2_-N) by the naphthylethylenediamine photometric method involved a detection limit of 0.01 μmol/L while determination of ammonia salt (NH_4_-N) with sodium salicylic acid used a detection limit of 0.03 μmol/L.

#### Chlorophyll Measurement

We took the filtered Chl *a* membrane samples out of the refrigerator, poured 20 mL of each of them into brown bottles, added 5 mL acetone solution with 90% concentration, and then put the bottles into −20°C in the dark for 24 h. We then used a fluorescence detector (Turner-Designs Trilogy TM) with the CHL-ANA module (Welschmeyer, 1994) to measure the fluorescence value of the sample.

#### Flow Cytometry Counting

After melting the frozen sample, part of each of the samples was combined with YG fluorescent (1 μm; Polysciences) pellets as an internal reference, and then analyzed with flow cytometry (Becton-Dickinson Accuri C6). Three taxa of picophytoplankton were divided and counted by the two-parameter graph of forward scatter light (SSC), orange fluorescence (FL2, 585 ± 40 nm), and red fluorescence (FL3, > 670 nm; FL4, 675 ± 25 nm) (Jiang et al., 2017). When measuring *Synechococcus*, their cells were distinguished from other picophytoplankton by the phycoerythrin pigments. *Synechococcus* emitted orange fluorescence; picoeucaryote had only strong red fluorescence but no orange fluorescence; *Prochlorococcus* had only weak red fluorescence. With the FL3 signal, *Prochlorococcus* cannot be divided because it overlaps with the background noise, which could result in greatly underestimating the cell abundance of *Prochlorococcus* (Gérikas et al., 2017), while the FL4 signal can better distinguish between *Prochlorococcus* and noise.

### Data Analysis

In our study, carbon biomass was estimated based on their abundance-to-carbon conversion factors: 250 and 53 fg C/cell for picocyanobacteria, *Synechococcus*, and *Prochlorococcus*, respectively, while picoeucaryote is 964 fg C/cell (Campbell et al., 1997). Then, the depth weighted average was used to estimate picophytoplankton carbon biomass in our study area, using the following formula:


(1) $$A=\left[\sum_{n}^{n+1}\frac{\left(A_{i}+A_{i+1}\right)}{2}\,\times \left(D_{i+1}-D_{i}\right)\right]/\left(D_{\text{MSL}}-D_{\text{S}}\right)$$

where A is the carbon biomass of picophytoplankton, n is the number of sampling layers; A*_*i*_* is the picophytoplankton biomass (μg C/L) at the sampling layer; D*_*i*_* is the depth at sampling layer *i* (m); D*_*MSL*_* is the maximum sampling layer (m), and D*_*S*_* is the surface sampling layer (5 m).

All average data were given as the values ± standard deviation (± SD). The surface and transects distribution of picophytoplankton cell abundance were mainly shown with Ocean Data View 4. The vertical distribution of cell abundance was processed with Origin 2018. Then, Arc Map was used to show a pie chart of the picophytoplankton carbon biomass percentage. To assess the corresponding relationship between the explanatory variables (cell abundance) and response variables (e.g., depth, temperature, salinity, nutrient, and other parameters), canonical correspondence analysis (CCA) and Pearson’s correlation coefficients were used to explore their relationship. In all tests, statistical significance was accepted at *p* < 0.05. Picophytoplankton abundance was log_10_-transformed to improve the normality of the data for statistical analysis. Unless otherwise stated, picophytoplankton abundance and biomass used for presenting the spatial distribution are expressed as the depth-weighted averages (calculated by dividing the trapezoidal integration of measured values for each variable by the maximum sampling depth) because *Prochlorococcus* and picoeucaryote abundances were relatively low in the surface layer. These depth-weighted averages, which involved a series of abundance variations with depth, were better suited for our distributional patterns than were discrete sample data.

## Results

### Hydrography and Nutrients

**FIGURE 2:**
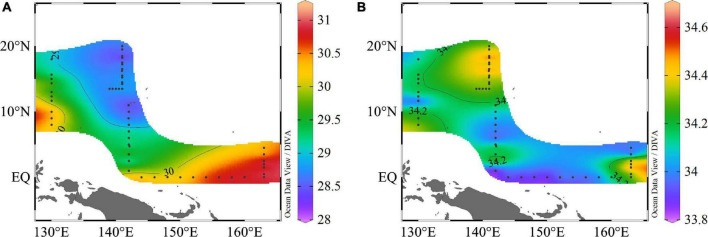
Spatial variation in surface **(A)** temperature (°C) and **(B)** salinity in the WPO.

During the period of our research, the study area encountered a strong, warm event. In general, the hydrographic conditions exhibited obvious horizontal and vertical variations. The surface temperature was higher than 28°C with salinity above 33 in the WPO. The surface temperature varied from 28.24 to 31.02°C, with an average of 29.58 ± 0.85°C. With the exception of the EQ and 165°E transect, there were high temperatures at 130°E, 8°N∼10°N; however, the lower temperature zone was confined to the 142°E transect, and temperature gradually decreased as the latitude increased. The surface salinity varied from 33.83 to 34.68, with an average of 34.19 ± 0.19; the salinity distribution was different to that of temperature. The area at 142°E, 15°N∼19°N was higher, and a high salinity region was also observed at 165°E, 1°N∼2°N, but the EQ transect was lower (Figure 2).

**FIGURE 3:**
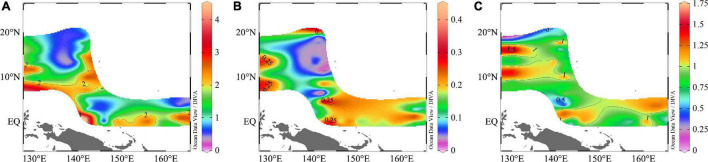
Spatial distributions of **(A)** DIN; **(B)** DIP; **(C)** DSi (μmol/L) in the WPO.

Nutrients concentrations were very low, and the surface concentrations were much lower than in other seas (e.g., South China Sea, Bay of Bengal) (Wei et al., 2020), especially phosphate concentrations. Because of the absorption and consumption of plankton, which cannot be supplemented in time, the nutrient concentrations were low. Apart for some zones, where coastal upwelling brought the underlying nutrients to the surface layer, most of the nutrients were under the detection limit. The ranges of DIN, DIP, and DSi concentrations were 0.13–4.46, 0.03–0.44, and 0.09–1.74 μmol/L, respectively, and the average value was 1.64 ± 0.95, 0.14 ± 0.09, and 0.91 ± 0.31 μmol/L for DIN, DIP, and DSi, respectively (Figure 3).

**FIGURE 4:**
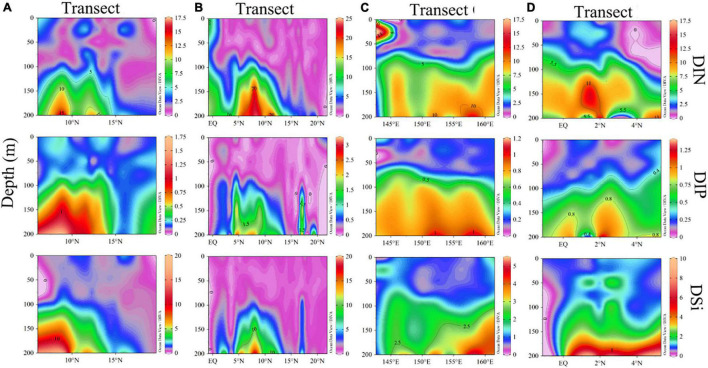
Section profile for concentrations of DIN, DIP, and DSi (μmol/L) along **(A–D)** transects.

We then compared the nutrients concentrations of four transects and found that the DIN and DIP concentrations of the EQ transect were higher, and DSi concentration of the 130°E transect was higher. Analysis of the nutrients profiles revealed that nutrients concentrations were relatively low in the upper layer, but increased rapidly from 100 to 200 m. Due to the pronounced stratification, nutrients were generally consumed by plankton within the upper 100 m. It can be seen from Figure 4 that there was a deep nutrient-cline at ∼100 m. Notably, there were high nutrients zones around the 8°N at a depth of 150∼200 m.

### Variability in Abundance and Biomass of Picophytoplankton Communities

Obvious variability in the picophytoplankton community was observed among sampling stations. In the community, *Prochlorococcus* generally dominated picophytoplankton abundance in our study region, followed by *Synechococcus* and picoeucaryote Picophytoplankton abundance had obvious vertical variation, which can usually occur in oligotrophic oceans with prominent stratification.

**FIGURE 5:**
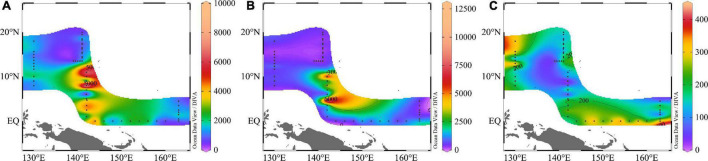
Spatial distributions of surface abundance of **(A)** *Synechococcus*; **(B)** *Prochlorococcus*; **(C)** Picoeucaryote (cells/mL).

**FIGURE 6:**
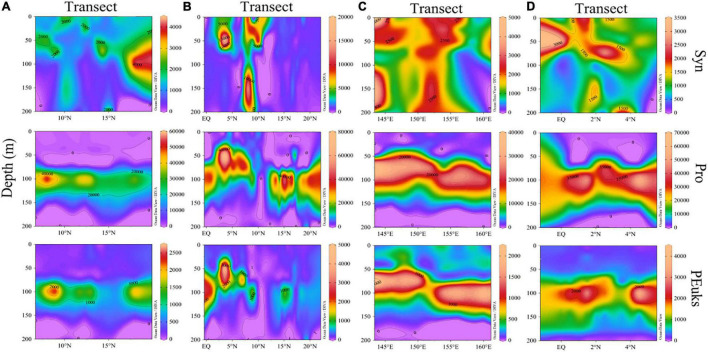
Section profile of abundances of picophytoplankton along **(A–D)** transects (cells/mL).

**FIGURE 7:**
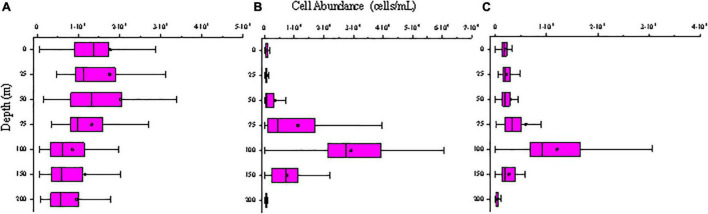
Vertical distribution of picophytoplankton abundance (cells/mL) in the study area of **(A)** *Synechococcus*; **(B)** *Prochlorococcus*; **(C)** Picoeucaryote.

*Synechococcus* was less abundant on average than *Prochlorococcus*, but its abundance was substantially more variable. Surface average abundance of *Synechococcus* was (1.87 ± 1.71) × 10^3^ cells/mL (ranging from 47.62 to 9.79 × 10^3^ cells/mL). Surface distribution was concentrated at the 142°E transect (Figure 5). From section distribution analysis, we concluded that *Synechococcus* was distributed irregularly (Figure 6). In transect A (transect 130°E), the cell abundance in the surface layer was higher than that in the bottom layer. Specifically, at station E130-1 (130°E, 18°N), there was a high value at 100 m. In transect B, there were areas of high cell abundance areas at depths of 50 m and 150∼200 m, at stations E142-6A (142°E, 3.5°N), E142-10 (142°E, 8°N), E142-11 (142°E, 9°N), and E142-12 (142°E, 10°N). In contrast to the A, B, and D transects, in the C transect a wide distribution of *Synechococcus* was still detected at more than 100 m depth. The cell abundance of *Synechococcus* in the D transect was the lowest. In terms of the vertical distribution of *Synechococcus* the cell abundance of *Synechococcus* was higher in the upper 50 m and decreased with the increasing depth below 75 m (Figure 7).

*Prochlorococcus* was the most abundant genus in the picophytoplankton community. Surface average abundance of *Prochlorococcus* was (1.02 ± 1.87) × 10^3^ cells/mL (ranging from 1.36 × 10^2^ to 1.12 × 10^4^ cells/mL). Highest surface cell densities of *Prochlorococcus* were found at the 142°E transect, which coincided with that of *Synechococcus* (Figure 5). The section distribution of *Prochlorococcus* had a certain regularity (Figure 6). Lower cell abundance appeared in the surface layer and at depths between 50 and 150 m. Cell abundance was highest in the 165°E transect. In terms of the vertical distribution of *Prochlorococcus*, cell abundance was higher in the layer of 75∼150 m; lower abundance appeared in the upper 25 and 200 m (Figure 7).

The cell abundance of the Picoeucaryote was lower than that of *Synechococcus* and *Prochlorococcus*, and not all stations could detect the distribution of picoeucaryote. The surface average abundance of picoeucaryote was (1.87 ± 0.88) × 10^2^ cells/mL (ranging from 0 to 4.11 × 10^2^ cells/mL). Its surface distribution was concentrated on the 130°E and EQ transects (Figure 5). Its section distribution coincided with the that of *Prochlorococcus*, at a depth of 50∼150 m (Figure 6). Cell abundance was the highest in the 165°E transect. In terms of the vertical distribution of picoeucaryote, cell abundance was higher in the layer up to 100 m (Figure 7).

**FIGURE 8:**
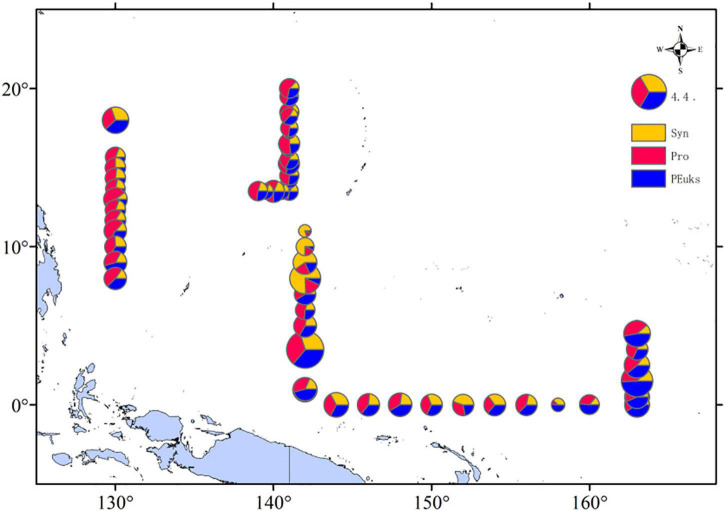
Spatial distributions of depth-weighted average carbon biomass (mg C/L) of *Synechococcus*; *Prochlorococcus*; Picoeucaryote.

According to the picophytoplankton cell abundances we measured, we roughly estimated the carbon biomass of the picophytoplankton community. *Prochlorococcus* was the main contributor to picophytoplankton carbon biomass, making up 41% of the total picophytoplankton carbon biomass in the WPO. The picoeucaryote was always a minor component of picophytoplankton total abundance, but it contributed significantly to carbon biomass, representing on average 35% of the carbon biomass, compared to 24% for *Synechococcus*. We then analyzed the percentage of these three picophytoplankton groups at each station by depth-weighted carbon biomass, and found that the carbon biomass distributed varied, at stations E142-6A (142°E, 3.5°N), E142-10 (142°E, 10°N), and E165-4.5 (163°E, 1.5°N) were higher (Figure 8).

### Concentrations of Pico-Sized Chl *a*

**FIGURE 9:**
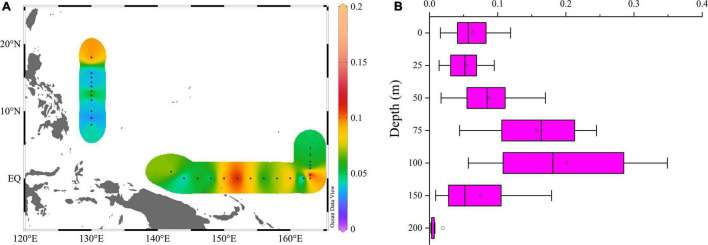
Horizontal **(A)** and vertical **(B)** distributions of pico-Chl *a* (μg/L).

The Pico-sized Chl *a* (Pico-Chl *a*) concentration varied from 0.001 to 0.353 μg/L, with mean of 0.11 ± 0.04 μg/L. A high pico-Chl *a* concentration was found around the equator and 18°N stations, and it was low at the 8∼13°N and (162°E, 0) stations (Figure 9A). The vertical profiles of Chl *a* showed a Gaussian shape. Chl *a* changed only slightly within the mixed layer, but below the mixed layer there was a deep chlorophyll maximum layer (DCM). The depth of the pico-Chl *a* maximum layer occurred at approximately 100 m, which was extremely similar to the vertical distribution of picophytoplankton cell abundance (Figures 7, 9B). pico-Chl *a* concentrations increased from the surface to 100 m, peaking at 100 m and then decreasing, with the lowest concentration occurring at 200 m.

### The Variation of Carbon to Chlorophyll *a* Ratio (C:Chl *a*) of Picophytoplankton

**FIGURE 10:**
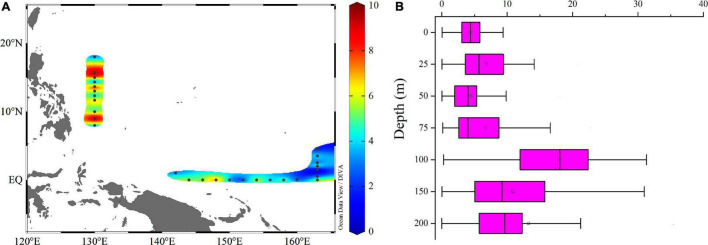
Horizontal **(A)** and vertical **(B)** profiles of C:Chl *a* ratio.

Changes in the ratio of C:Chl *a* were associated with substantial vertical and spatial variations in the picophytoplankton community and pico-Chl *a* concentration. Since the pigment for photosynthesis in *Synechococcus* is phycoerythrin, not chlorophyll, *Synechococcus* cannot be taken into account when assessing the C:Chl *a* ratio. Thus, carbon was calculated as the sum of *Prochlorococcus* and the picoeucaryote (which are derived from *Prochlorococcus* and picoeucaryote-specific flow cytometry counts). Horizontal distributions of C:Chl *a* (Figure 10A) suggested that areas with a higher C:Chl *a* ratio occurred at stations close to the Philippines. From the vertical change of the C:Chl *a* ratio with water depth (Figure 10B), it can be seen that the ratio was higher at depths of 100∼150 m. With increasing water depth, the photosynthetically available radiation and seawater temperature both decreased and the photosynthesis rate of the piocphytoplankton decreased significantly. Decreasing chlorophyll synthesis rates is a way for piocphytoplankton to reduce their energy consumption. On the other hand, piocphytoplankton were continuously accumulating carbon, which led to the larger C:Chl *a* ratio at 100∼150 m.

## Discussion

### Significance of *Prochlorococcus* Among the Picophytoplankton Community in the Western Pacific Ocean

Studies have shown that as the oligotrophic nature of the marine system increases, the percentage of *Prochlorococcus* as a fraction of the total procaryotes population would increase (Yang and Jiao, 2004). In the typical oligotrophic oceans, the ratio of *Prochlorococcus* to *Synechococcus* is between 50 and 200 (Campbell et al., 1988). However, we calculated the cell abundance ratio of *Prochlorococcus* to *Synechococcus* to be below 20 in the WPO (Supplementary Figure 1A), and the result was related to the distribution of *Prochlorococcus* and *Synechococcus* (Figure 5). In terms of the picophytoplankton community structure, we derived different results from those reported previously. Although the Pacific Ocean is an oligotrophic ocean, there are areas where the oligotrophy is less severe than it is in the Indian Ocean (Campbell et al., 1988). In terms of carbon biomass, *Prochlorococcus* was smaller in cell diameter, but accounted for about half of the carbon biomass of the picophytoplankton community. Moreover, *Prochlorococcus* plays an significant role in global marine primary productivity. The cell abundance ratio of *Prochlorococcus* to *Synechococcus* was also an ideal parameter for evaluating hydrological characteristics (Campbell et al., 1988). Generally, where the ratio was high, temperature and salinity was also high. As suggested by the distribution of temperature and salinity (Figure 2), we speculated that the area with a high ratio indicated different picophytoplankton community structures which might be affected by the hydrological environment. Another fascinating finding was that the negative correlation between *Prochlorococcus* and *Synechococcus* was observed in the WPO (Supplementary Figure 1B), similarly to the case in other oceans. The seasonally and spatially inverse relationship of cell abundance of these two groups in the North Pacific was first reported by Olson et al. (1990), while Jiao and Yang (1999) noted the negative correlation between *Prochlorococcus* and *Synechococcus* in the East China Sea. Moreover, these patterns are also present in the tropical western Pacific from 20°S to 7°N (Blanchot and Rodier, 1996). However, the inconsistent relationship between *Prochlorococcus* and *Synechococcus* was also observed in other studies, such as in the Nansha Island area (Yang and Jiao, 2004) and the winter Mediterranean Sea (Vaulot et al., 1990). It was concluded that the regulation mechanisms at a large spatial scale and long-time scale might be different from those for small spatial and time scales (Brian et al., 1996). We speculate that an additional reason was the environmental adaptability of *Prochlorococcus*, the differentiation of *Prochlorococcus* ecotypes and sub-ecotypes reflected its continual adaptation to the changeable marine environment (Yan et al., 2020).

As a result of global warming, the thermocline in the oceans has become deeper, and the photic zone may become more oligotrophic due to a lack of supply of nutrients. As a result, seawater stratification will become more intense and severe. Even worse, marine phytoplankton biomass was predicted to decline, due to the expansion of nutrient-depleted ocean regions. Studies have inferred that future warmer ocean conditions can lead to elevated biomass in regions that were already dominated by picophytoplankton (Flombaum et al., 2020). These conditions are conducive to the bloom of *Prochlorococcus*, thus the global abundance of *Prochlorococcus*, which require relatively lower nutrient concentrations, may increase by 30%, and have a tendency to expand from low latitudes toward high latitude oceans. Twelve ecotypes of *Prochlorococcus* have been discovered (Yan et al., 2020). Perhaps the high-temperature adapted ecotype (HLII, high-light-adapted II) will spread to high-latitude regions, while the low-temperature adapted ecotype (HLI, high-light-adapted I) will expand its distribution toward lower latitude oceans (Biller et al., 2015). At present, the response of *Prochlorococcus* subspecies to global climate change is unpredictable and worthy of further exploration (Yan et al., 2020).

### Relationship Between C:Chl *a* and Environmental Factors

Our results suggest that environmental factors have a possible effect on the variability of C:Chl *a* (Supplementary Figure 2). Actually, there was a strong negative association between water temperature and C:Chl *a* and a good positive correlation between C:Chl *a* and depth, salinity, and nutrient. These results are consistent with previous observations (Finenko et al., 2003). Finenko et al. (2003) reviewed the effects of temperature and nutrient limitation on the C:Chl *a* ratio of 36 micro-algae species from seven taxonomic groups. They found that at constant light levels, the C:Chl *a* ratio decreased exponentially with increasing temperature. Further, at steady state, the nutrient consumption rate of micro-algae was proportional to their growth rate. In addition to the above factors, the C:Chl *a* ratio also varied with the phytoplankton group, being lowest for the larger diatom cells and highest for smaller species such as *Prochlorococcus* sp. (Sathyendranath et al., 2009). Also, there was a more variable contribution to the particulate carbon from material other than picophytoplankton in oligotrophic waters, whereas the relationship between particulate carbon and picophytoplankton carbon would be tighter at higher Chl *a* concentrations (Sathyendranath et al., 2009). Strickland (1960) discovered the limitations of C:Chl *a* ratio estimations 50 years ago. Although several models had been developed, none of them were fast, accurate, and simple enough to measure the carbon content of marine phytoplankton.

### Environmental Factors Influencing the Distribution of the Picophytoplankton Community

WPO has a complex hydrological environment, characterized by high temperatures and an oligotrophic water body. Our study covered many representative regions such as the North Equatorial Current and the Western Pacific Warm Pool. According to the distribution of surface temperature and salinity (Figure 2), the North Equatorial Current and Kuroshio played an important role in these environments (Nitani, 1972). The North Equatorial Current moved from the east to the west, with the characteristics of high temperature and low salinity, which caused higher temperatures and lower salinity near the equator. After arriving at the west boundary, the North Equatorial current was hindered by topography, and the current was divided into the Kuroshio and Mindanao currents. Kuroshio was characterized by high salinity, which resulted in high salinity at 10°N (Hu and Wang, 2011). In addition, we speculated that the transportation of the Mindanao Current and the support of the semi-permanent Mindanao Eddy might resulting in higher nutrients concentrations around the 8°N at a depth of 150∼200 m (Figure 4; Hu and Cui, 1991). These changing environments had a great impact on the hydrology, meteorology, and biological community in the ocean.

Picophytoplankton community structure in the WPO displayed spatial variations in response to physical variables. Many literatures have recorded the importance of temperature on the abundance and distribution of picophytoplankton in various oceans (Agawin et al., 2000; Moisan et al., 2010; Chen et al., 2014). The data from our study showed that *Synechococcus* and *Prochlorococcus* have roughly the same distribution trends, concentrating in the 28∼32°C regions. This result suggests that these two groups are better adapted to grow in warmer waters. The findings of Kuosa (1990) and Li (1998) supported our conclusion. Kuosa (1990) found that *Synechococcus* did not grow at temperatures below 10°C, while Li (1998) found that the abundance of *Synechococcus* was directly related to temperatures below 14°C. Marcus and Bryant (2012) used global transcriptome profiling to perform a comparative and integrated study of global changes in *Synechococcus* grown at different hydrological characteristics. They found that *Synechococcus* grown at lower temperatures was slightly limited by CO_2_. Compared to standard culture conditions, low temperatures could reduce the transcript level of *nblA* in *Synechococcus* cells. which would make phycobiliprotein sensitive to proteolytic degradation (Baier et al., 2004; Karradt et al., 2008). When the growth environment was bad, *nblA* could be transcribed heavily to promote the release and utilization of reduced carbon and nitrogen (Collier and Grossman, 1992). In addition, the transcript level of gene encoding enzymes involved in carbohydrate degradation also slightly decreased (Marcus and Bryant, 2012). Compared with *Synechococcus*, *Prochlorococcus* had a slightly slower response to temperature changes. For *Prochlorococcus*, there were many ecotypes that could be used to adapt to different temperature changes. HLI and HLII were distributed in the open ocean between 45°N and 45°S, and they mainly thrive in the upper or middle euphotic zone, accounting for more than 90% of all *Prochlorococcus* abundance (Johnson et al., 2006). On a global scale, the abundance of HLII was much higher than that of HLI, and the HLII ecotype can grow at higher temperatures (> 28°C) (Johnson et al., 2006). On the other hand, temperature was the main environmental factor affecting the distribution and quantity of *Prochlorococcus* in the field. Although the high abundance of *Prochlorococcus* was always associated with high temperature, when the culture temperature was increased from 21to 29°C, one major beneficiary was heterotrophic bacteria, and the growth of *Prochlorococcus* was negatively affected (Chisholm et al., 1988).

The surface layer is not the most suitable living conditions for *Prochlorococcus*. The surface layer had strong light radiation, high temperatures and low concentrations of nutrients. With the increased water depth, light intensity and temperature gradually decreased, while nutrient concentrations increased rapidly, resulting in a large gradient of environmental factors (Biller et al., 2015). From the perspective of genomics, the highlight-adapted (HL) group contained highly compressed genomes, which enabled them to grow with less nutrients and energy, increasing their competitive advantage in the oligotrophic environments (Partensky and Garczarek, 2010). The low-light-adapted (LL) group had streamlined genomes, resulting in a stronger light absorption capacity than that of the large phytoplankton by reducing their cell volume in low light environments (Yan et al., 2020). From energetic cost-benefit considerations, the shorter-wavelength photosynthetically active radiation dominated at depth in most water bodies, Chl *b* (and *c*) had a higher specific absorption coefficient in the blue region of the spectrum and these chromophores were capable of wavelength-specific absorption characteristics in very small cells (Raven, 1996). The photosynthetic pigmentation of *Prochlorococcus* means that it had a more effective use of Fe than did *Synechococcus*, making the Chl *b*-containing picophytoplankton potentially very effective in growing in low-Fe and low-light habitats.

The cell abundance of picophytoplankton was probably affected by their growth conditions. In addition to the effects of ocean currents and temperature mentioned above, light conditions and nutrients concentrations also had a certain impact on them. Unfortunately, due to the limitation of field sampling, we did not obtain the light intensity parameters, and can only roughly speculate on them based on water depth. Therefore, we focused on the impact of environmental factors (depth, seawater salinity, nutrient) on the cell abundance and distribution of picophytoplankton. The environmental factors dataset was analyzed to identify which environmental variables were the main factors affecting the biogeographic variation of picophytoplankton (Supplementary Figure 3). Abundances of *Prochlorococcus* were significantly positively correlated with salinity and depth. Picoeucaryote was positively correlated with temperature. Nutrients concentrations were the potential bottom-up factors of environmental impact factors that drove picophytoplankton community dynamics. Results of CCA results showed that *Prochlorococcus* and *Synechococcus* were positively correlated with nutrients (DIN, DIP, and DSi), suggesting that *Prochlorococcus* and *Synechococcus* were favored by the optimal nutrients concentrations, but no significant correlations were found between picoeucaryote and nutrients.

Under the oligotrophic conditions, small size is unique to picophytoplankton and their resulting high surface-to-volume ratio has significant adaptive advantages with regards to nutrient uptake (Chisholm, 1992). Partensky et al. (1999) proposed the hypothesis that there are different ecotypes of *Prochlorococcus* adapted to grow on different nutrient sources. While nitrogen was considered a major limiter in many marine areas, recent evidence has suggested that phosphorus is also restrictive, in areas such as the Mediterranean and the Sargasso Sea (Thingstad and Rassoulzadegan, 1995; Cotner et al., 1997). *Prochlorococcus* also has the ability to thrive at very low phosphorus concentrations, which is limited only when the concentration of P-PO_4_^3–^ is below 30 nM (Palenik, 1994). Notably, *Prochlorococcus* has a *pstS* gene encoding a phosphate-binding protein, which is expressed only in the absence of phosphorus and may play a role in its oligotrophic adaptation. Motile marine *Synechococcus* strains have been reported (Toledo et al., 1999). These mobile marine strains of *Synechococcus* are not normally found in nutrient-rich coastal areas, but rather in nutrient-poor areas where nitrogen is limited. Research has shown that *Synechococcus* has chemotaxis to various nitrogen compounds. We believed that the ability to detect and seek out areas of enriched nutrients might provide an ecological advantage to them. For picoeucaryote, environmental heterogeneity was closely related to picoeucaryote groups variations, and research suggests that active currents promoted the dispersal of picoeucaryote in the marine system (Wu et al., 2017).

On the other hand, the distribution of *Prochlorococcus*, *Synechococcus*, and the picoeucaryote at different water depths varied, and only *Synechococcus* had an obvious negative correlation with depth. This indicates that light irradiance is a key limiting factor for the temporal-spatial distributions of picophytoplankton. In general, the light requirement of *Synechococcus* was higher than that of *Prochlorococcus*; thus, the distribution of the two groups had obvious complementarity in the distribution of the photic zone, that is, *Synechococcus* was mainly distributed on the surface layer, and *Prochlorococcus* was widely distributed in the sub-surface and bottom layer. Some studies have shown that *Prochlorococcus* was inhibited by strong light, especially UVR; as a result, *Prochlorococcus* was better adapted to capture the blue wavelengths that permeated the depths of water (Goericke et al., 2000; Hawco et al., 2020). To sum up, picophytoplankton were most abundant in oligotrophic open waters, but they were not limited to this habitat. However, our research was not precise enough. We could only roughly describe the impact of the marine environment on the growth and distribution of picophytoplankton and could not uncover a close relationship between picophytoplankton physiological characteristics and biogeographic distribution. More relevant data are needed to further explore this relationship.

## Data Availability Statement

The original contributions presented in the study are included in the article/Supplementary Material, further inquiries can be directed to the corresponding author/s.

## Author Contributions

JS conceived and designed the experiments. FW wrote the manuscript and analyzed the data. FW and GZ carried out the sample collection and determined the environmental parameters. FW, JS, YW, GZ, and LZ contributed to manuscript development and revisions. All authors read and approved the final manuscript.

## Conflict of Interest

The authors declare that the research was conducted in the absence of any commercial or financial relationships that could be construed as a potential conflict of interest.

## Publisher’s Note

All claims expressed in this article are solely those of the authors and do not necessarily represent those of their affiliated organizations, or those of the publisher, the editors and the reviewers. Any product that may be evaluated in this article, or claim that may be made by its manufacturer, is not guaranteed or endorsed by the publisher.
