# Supplementary material for: Picophytoplankton in the West Pacific Ocean: A Snapshot
Source: Front Microbiol. 2022 Mar 22;13:811227. doi: 10.3389/fmicb.2022.811227 (PMC8981306; doi:10.3389/fmicb.2022.811227)

**Supplemental Material**

**Figure S 11.** The ratio of cell abundance of *Prochlorococcus* to *Synechococcus* (a) and their relationship (b).

**Figure S 12.** Canonical correspondence analysis of the C:Chl *a* ratio (*Prochlorococcus* & Picoeucaryote) and environmental parameters in the WPO

**Figure S 13.** Canonical correspondence analysis of nutrient variables with picophytoplankton groups.

**Figure. S11**

**Figure. S12**


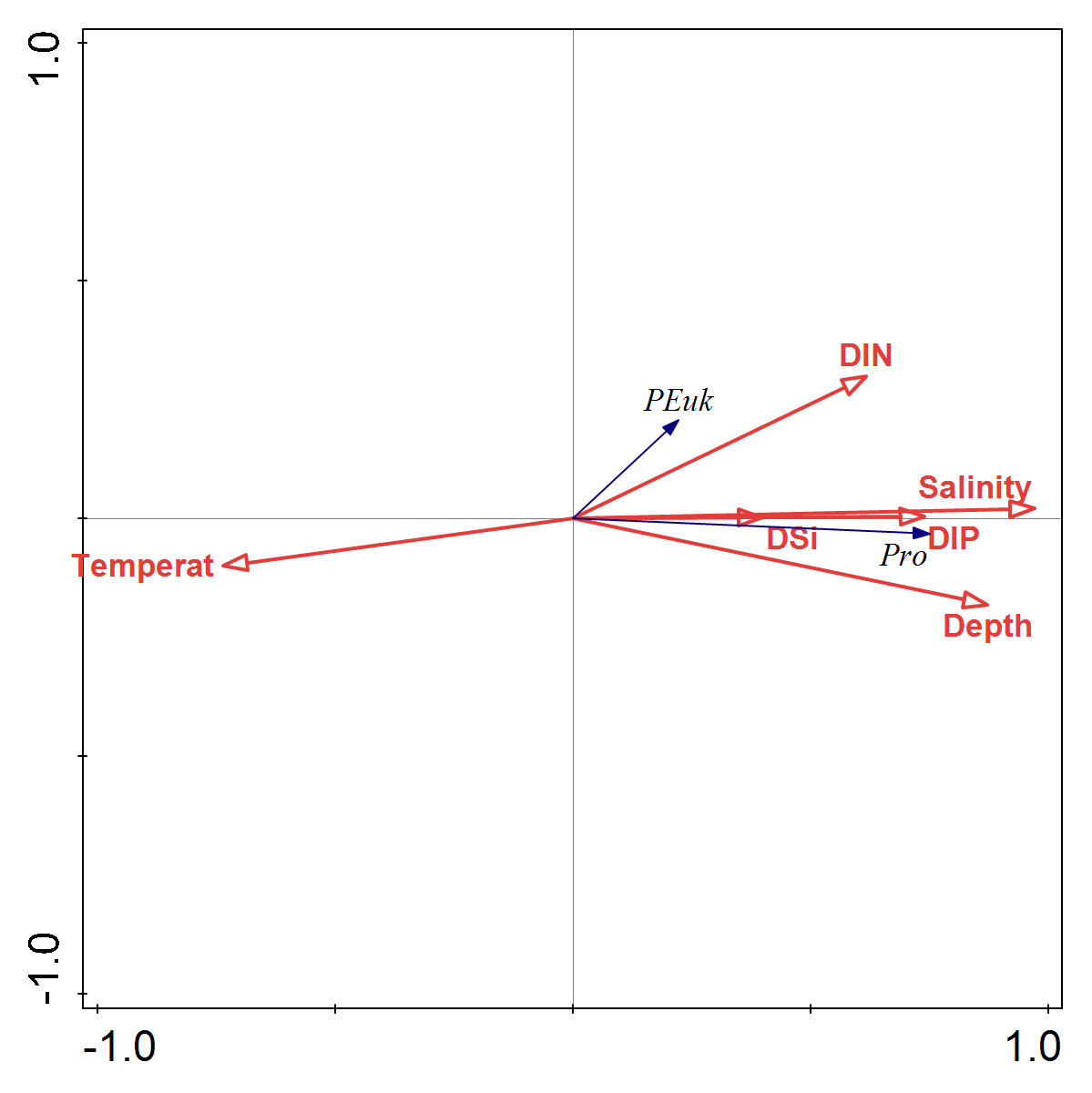


**Figure. S13**


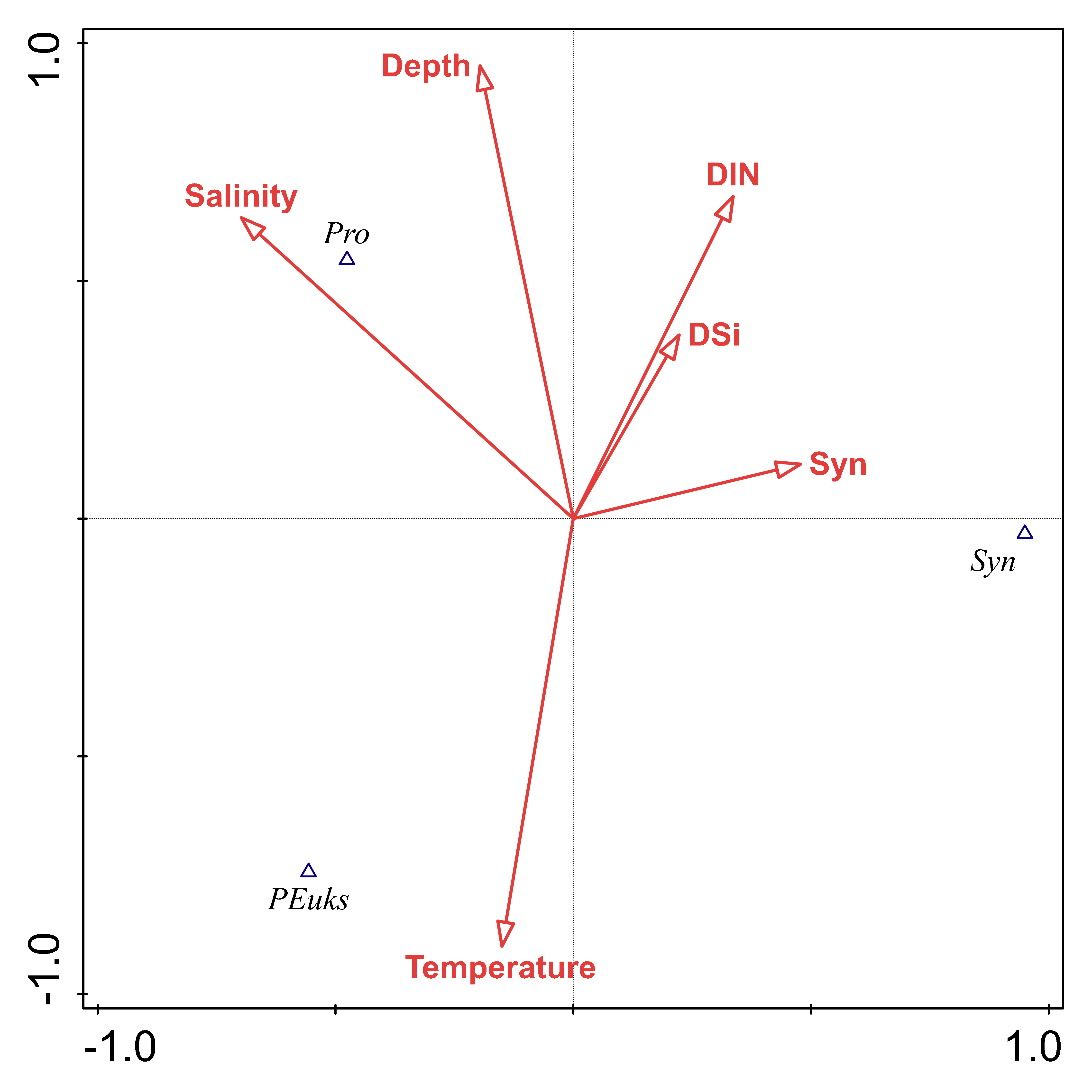

Supplement: Supplementary file 1 [file Data_Sheet_1.docx]
